# A 2D video-based assessment is associated with 3D biomechanical contributors to dynamic knee valgus in the coronal plane

**DOI:** 10.3389/fspor.2024.1352286

**Published:** 2024-03-15

**Authors:** Ashley Erdman, Alex Loewen, Michael Dressing, Charles Wyatt, Gretchen Oliver, Lauren Butler, Dai Sugimoto, Amanda M. Black, Kirsten Tulchin-Francis, David M. Bazett-Jones, Joseph Janosky, Sophia Ulman

**Affiliations:** ^1^ Movement Science Lab, Division of Sports Medicine, Scottish Rite for Children, Frisco, TX, United States; ^2^ Department of Orthopedics, Joe DiMaggio Children’s Hospital, Hollywood, FL, United States; ^3^ Department of Orthopaedic Surgery, University of Texas Southwestern Medical Center, Dallas, TX, United States; ^4^ Sports Medicine & Movement Laboratory, School of Kinesiology, Auburn University, Auburn, AL, United States; ^5^ Department of Rehabilitation, Nicklaus Children’s Hospital, Miami, FL, United States; ^6^ Faculty of Sport Sciences, Waseda University, Tokyo, Japan; ^7^ Sports Medicine Division, The Micheli Center for Sports Injury Prevention, Waltham, MA, United States; ^8^ Centre for Healthy Youth Development Through Sport, Department of Kinesiology, Faculty of Applied Health Sciences, Brock University, St. Catharines, ON, Canada; ^9^ Department of Orthopedic Surgery, Nationwide Children’s Hospital, Columbus, OH, United States; ^10^ Department of Exercise and Rehabilitation Sciences, College of Health and Human Services, The University of Toledo, Toledo, OH, United States; ^11^ Sports Medicine Institute, Hospital for Special Surgery, New York, NY, United States

**Keywords:** anterior cruciate ligament injury, injury prevention, motion capture, video analysis, qualitative movement analysis

## Abstract

**Introduction:**

Adolescent athletes involved in sports that involve cutting and landing maneuvers have an increased risk of anterior cruciate ligament (ACL) tears, highlighting the importance of identifying risky movement patterns such as dynamic knee valgus (DKV). Qualitative movement screenings have explored two-dimensional (2D) scoring criteria for DKV, however, there remains limited data on the validity of these screening tools. Determining a 2D scoring criterion for DKV that closely aligns with three-dimensional (3D) biomechanical measures will allow for the identification of poor knee position in adolescent athletes on a broad scale. The purpose of this study was to establish a 2D scoring criterion that corresponds to 3D biomechanical measures of DKV.

**Methods:**

A total of 41 adolescent female club volleyball athletes performed a three-task movement screen consisting of a single-leg squat (SLS), single-leg drop landing (SLDL), and double-leg vertical jump (DLVJ). A single rater scored 2D videos of each task using four criteria for poor knee position. A motion capture system was used to calculate 3D joint angles, including pelvic obliquity, hip adduction, knee abduction, ankle eversion, and foot progression angle. Receiver operating characteristic curves were created for each 2D scoring criterion to determine cut points for the presence of movement faults, and areas under the curve (AUC) were computed to describe the accuracy of each 2D criterion compared to 3D biomechanical data.

**Results:**

3D measures indicated knee abduction angles between 2.4°–4.6° (SD 4.1°–4.3°) at the time point when the center of the knee joint was most medial during the three tasks. AUCs were between 0.62 and 0.93 across scoring items. The MEDIAL scoring item, defined as the knee joint positioned inside the medial border of the shoe, demonstrated the greatest association to components of DKV, with AUCs ranging from 0.67 to 0.93.

**Conclusion:**

The MEDIAL scoring criterion demonstrated the best performance in distinguishing components of DKV, specifically pelvic obliquity, hip adduction, ankle eversion, and foot progression. Along with the previously published scoring definitions for trunk-specific risk factors, the authors suggest that the MEDIAL criterion may be the most indicative of DKV, given an association with 3D biomechanical risk factors.

## 1 Introduction

The prevalence of anterior cruciate ligament (ACL) tears in adolescent athletes is rising as year-long sport participation becomes more common (1). Over 20 years, the rate of ACL tears among adolescent athletes increased by 2.3% annually, with a higher incidence of injuries occurring among females (1–3). Compared to males, females have been shown to exhibit distinctive biomechanical and neuromuscular risk factors for ACL injury, including stiffer landings with reduced hip and knee flexion, and increased hip adduction (4–6). This increased risk, particularly in sports that involve cutting and landing maneuvers, highlights the importance of injury prevention through identification of risky movement patterns. While knee abduction is one component of injury risk, faulty movement patterns across multiple joints directly relate to ACL injury (7–10). Screenings for movement patterns associated with ACL injury mechanisms can be administered to identify athletes at risk. While various screening tools have been utilized for the identification of injury risk, there remains a lack of consensus and validation vs. more advanced movement assessment technology, such as motion capture (11–15). Therefore, a method for accurately identifying faulty movement patterns with more accessible 2D technology is needed.

The role of coronal knee position (knee abduction) and associated forces that impact the knee joint during running and landing tasks have been widely investigated as contributors to ACL injury. Previous studies have prospectively investigated three-dimensional (3D) biomechanical factors in participants that subsequently experienced an ACL injury and found that increased knee valgus angles and moments were predictors of ACL injury (10, 16). Therefore, numerous efforts have been made to develop screening tools for dynamic knee valgus (DKV) and loading on the knee using low-cost alternatives to 3D motion capture (11, 13, 14, 17–22). However, few validated tools have shown promise in discerning DKV. For instance, Padua et al. investigated the validity of the Landing Error Scoring System (LESS), a two-dimensional (2D) clinical tool used to assess movement patterns during a drop vertical jump task. They found that poorer LESS scores were associated with increased knee abduction measured simultaneously with 3D motion capture (23). While participants with poorer scores on the LESS were found to exhibit altered lower extremity kinematics, significant differences reported in peak knee abduction were less than 2°. This is a relatively small difference compared to the inherent measurement error of 3D motion capture (typically considered to be within 3°) and may lack clinical significance (24).

While 3D motion capture has long been considered the gold standard for assessing lower extremity biomechanics (10, 20, 25, 26), the inherent limitations of equipment cost and accessibility hinder its application on a large scale. Qualitative movement screening tools have been suggested for large scale testing, however, agreement between 3D measures of knee abduction and qualitative movement screening tools are varied (21, 27–32). Potential cross-talk with 3D methods can result in inaccurate measurement of knee abduction in tasks that involve increased amounts of knee flexion, resulting in established variability in agreement with 2D methods (33–35). Researchers have demonstrated that agreement between 2D frontal plane knee motion and 3D knee abduction angles differ widely depending on the task being evaluated (29). In prior work, the author team identified poor agreement with 3D knee abduction angle and 2D scoring criterion using a three-task movement screen (36). The use of 2D video analysis for measuring frontal plane knee kinematics is cautioned when accurate measures are desired, and further investigation into classifying frontal plane knee position based on easily discernible landmarks was warranted (29, 36). Contributions from motion at multiple joints, expressly hip adduction and internal rotation, knee abduction, tibial external rotation and anterior translation, and ankle eversion are considered predictors of ACL injury risk (37). Therefore, biomechanical contributions from the hip, knee and ankle may provide improved alignment with 2D visual assessments for movement faults at the knee.

While other visually derived definitions for DKV have been explored, there remains limited data on the validity of 2D screening tools to identify DKV as an ACL injury risk factor, specifically among female athletes (20, 28, 38–40). Since ACL injury risk has been found to vary by sport, sport-specific screenings are needed to more precisely identify injury risk factors (41–44). Female athletes, specifically adolescent volleyball players, are at an increased risk of ACL injury during sport-specific landing tasks, such as block jumps or jump attack maneuvers, due to stiff landing mechanics and medial knee collapse (6, 45). To our knowledge, no validated 2D movement screen exists to identify DKV specifically for female volleyball players. Determining an accurate 2D scoring criterion for DKV that more closely aligns with 3D biomechanical measures will allow for the identification of DKV as an ACL injury risk factor in adolescent athletes on a broad scale. Therefore, the purpose of this study was to establish a 2D scoring criterion that corresponds to 3D biomechanical measures of DKV. We hypothesized that a single 2D scoring criterion, based on four different 2D definitions of DKV, across a three-task movement screen, would indicate excellent concurrent validity compared to 3D biomechanical measures.

## 2 Materials and methods

### 2.1 Study design and participants

A convenience sample of 41 female participants (10–18 years) was recruited from local volleyball clubs for this cross-sectional study. Participants were excluded if they reported a recent musculoskeletal injury within the previous three months or were diagnosed with an orthopedic condition that would limit their ability to perform the required tasks. The study was approved by the University of Texas Southwestern Institutional Review Board (Approval ID #082010-134), and all participants provided informed assent/consent before participation.

### 2.2 Data collection

**Figure 1:**
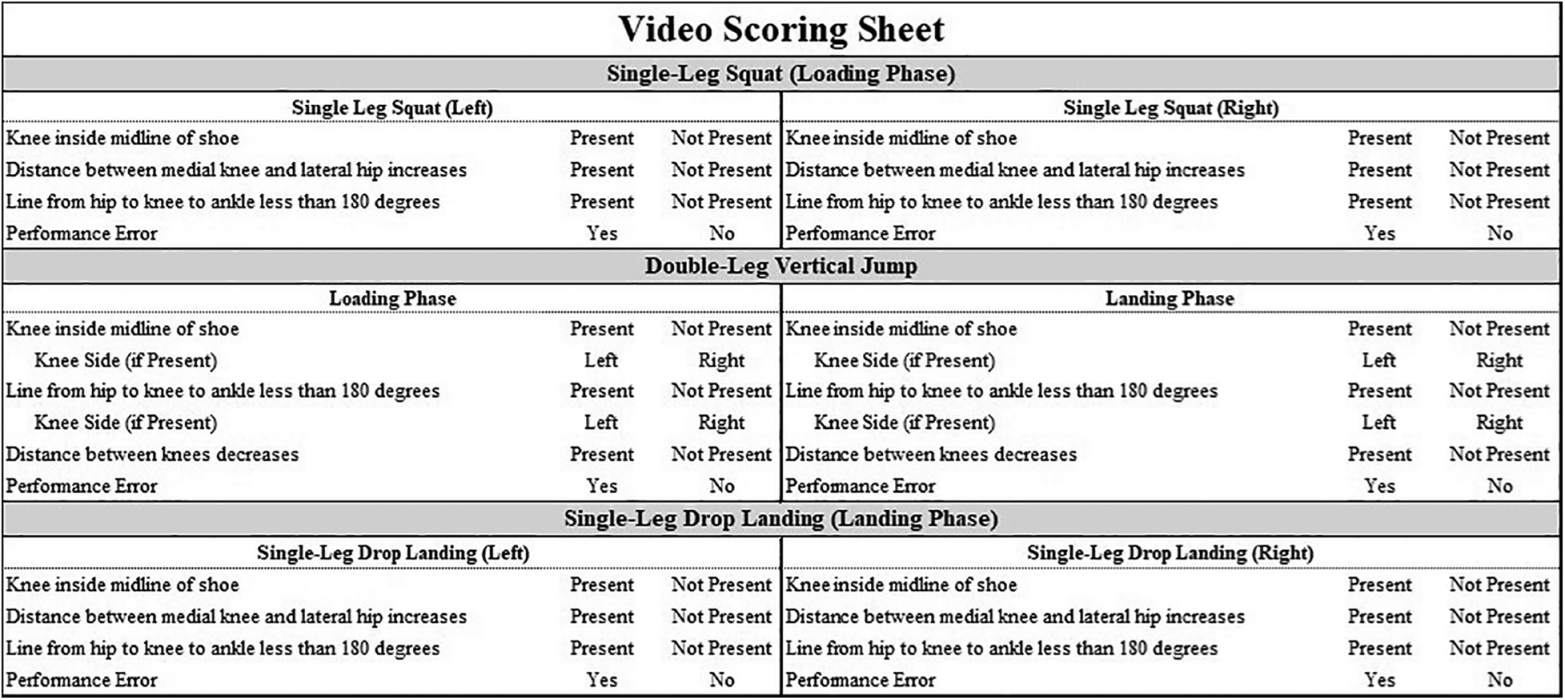
Marker set diagram. CLAV: clavicle notch, XP: xiphoid process, T1/T10: 1st and 10th thoracic vertebrae, ASIS: anterior superior iliac spine, SACR: sacrum, THC: rigid thigh cluster, LKN/MKN: lateral and medial femoral epicondyle, SHC: rigid shank cluster, LANK/MANK: lateral and medial malleolus, 5MET: 5th metatarsal head, TOE: 2nd metatarsal head, HEEL: midline of the hindfoot at approximately the same height as the TOE marker.

Participants were instrumented with 21 retroreflective markers placed bilaterally on bony landmarks along with rigid clusters placed on each thigh and shank segment (Figure 1). A 14-camera motion capture system (Vicon Motion System Ltd., Denver, Colorado, USA) was used to collect 3D kinematic data captured at 240 Hz while participants performed a three-task movement screen. Simultaneous video data were recorded at 60 frames per second with 1080p quality using a single video camera (Sony Cyber-shot DSC-Rx10, Tokyo, Japan) positioned 136 inches in front of the participant and 36 inches from the floor.

The three-task movement screen included a double-leg vertical jump (DLVJ), single-leg squat (SLS), and single-leg drop landing (SLDL) task. For the DLVJ, participants were instructed to jump as high as possible from a standing position, using their arms for momentum. For the SLS, participants began standing on one leg with the opposite leg bent behind them and hands on their hips. They were then instructed to squat as low as they could comfortably and return to the starting position. For the SLDL, participants were instructed to jump from a 31-cm plyometric box, land on one leg, and hold the landing for at least two seconds with hands on their hips. The SLS and SLDL were performed on each leg. Three practice trials were performed for each task to ensure participants were comfortable with the task and to confirm their understanding of the task instructions. Errors for each task were defined based on previous literature (11, 30, 46). A single attempt per task was collected, with the participant facing the video camera. A second attempt was granted if an error was observed during the first attempt and thus was determined to be unsuccessful (e.g., loss of balance, failure to stabilize upon landing). However, if the second attempt was also unsuccessful, their 2D data were not scored and they were removed from the analysis.

### 2.3 Injury risk factor assessment

**Figure 2:**
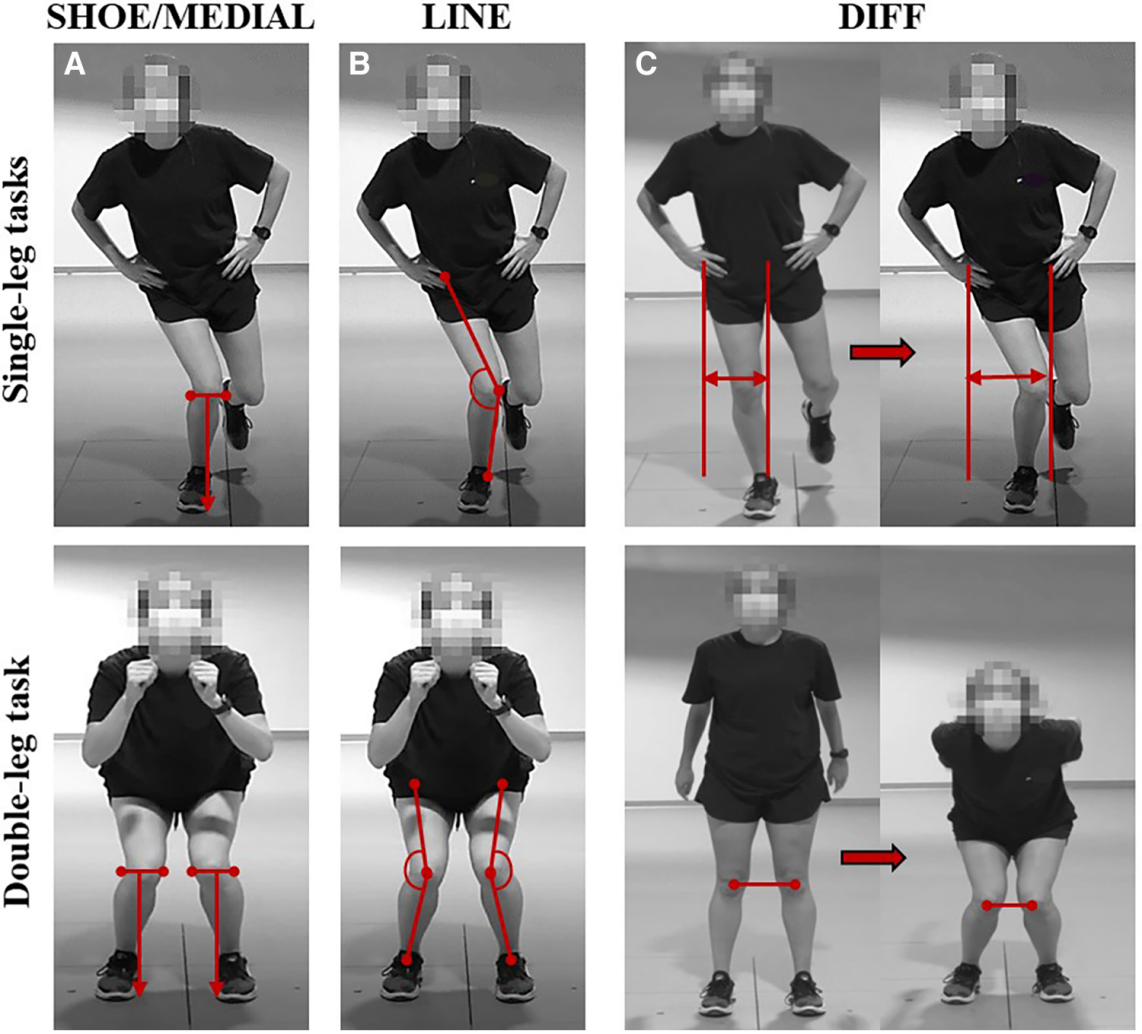
(**A**) SHOE: knee joint center inside the midline of the shoe. MEDIAL: Knee joint center inside the medial border of the shoe. (**B**) LINE: Angle from hip to knee to ankle is less than 180°. (**C**) DIFF: Distance between the medial knee and lateral hip increases (single-leg tasks, top row) or distance between the knee joint centers decreases (double-leg task, bottom row).

**Figure 3:**
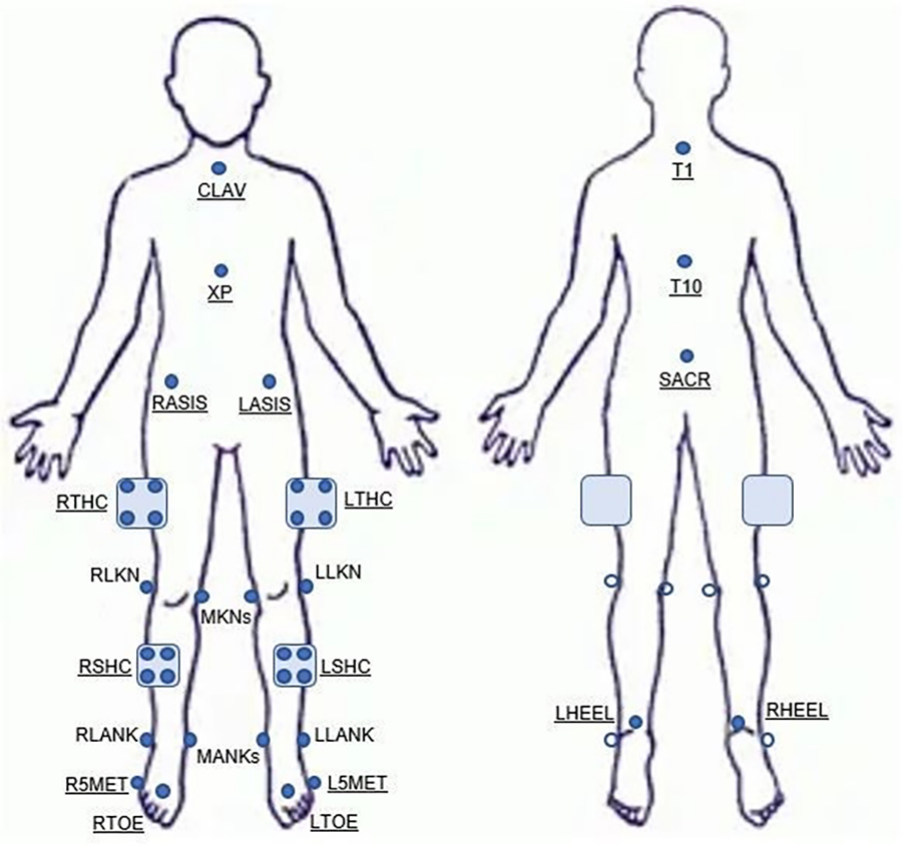
Video scoring sheet used to identify 2D movement faults per task.

Movement patterns were assessed by applying four different 2D scoring criterion that are currently used clinically and for screening purposes by our author team, and were derived from previous literature. The first criterion identified a movement fault as present if the center of the knee joint was inside the midline of the shoe (SHOE; Figure 2A). The second criterion identified a movement fault as present if the center of the knee joint was inside the medial border of the shoe (MEDIAL; Figure 2A). Both the SHOE and MEDIAL criteria characterized the knee position relative to the foot, which was derived from work that evaluated 2D thigh and tibia angles in relation to the knee over foot position (47). The third criterion identified a movement fault as present if the line from the center of the hip joint to the knee joint center (bisecting the thigh), to the middle of the ankle joint created an angle on the lateral side of the knee of less than 180° (LINE; Figure 2B). Previous studies have applied a similar technique of visualizing a line from the ASIS to the knee joint center and from the knee joint center to the ankle joint center (21, 40, 47–49). The fourth criterion identified a movement fault as present if the distance between vertical lines visualized on the lateral edge of the stance hip and the medial edge of the stance knee increased during single-leg tasks (DIFF; Figure 2C top) or if the distance between the knee joint centers decreased during the double-leg task (DIFF; Figure 2C bottom). The basis for the DIFF criterion was established from previous literature which assessed frontal plane knee displacement relative to the lateral edge of the hip (single-leg tasks) or knee separation distance (double-leg tasks) (39, 50). Recorded video data was reviewed and scored per the 2D scoring criterion for each task, and movement faults were identified and categorized as present or not present for each leg (Figure 3). Videos were viewed on a 17-inch laptop, using VLC media player (VideoLAN, Paris, France), which allowed for the reviewer to fast forward, rewind, pause and reduce playback speed when assessing for movement faults. Additionally, the use of a straight edge was permitted in order to assess alignment according to the criterion definitions.

For each task, phases of interest were identified in which the movement fault was assessed using each scoring criterion. Phases of movement were defined for the loading and landing phase of the DLVJ (DLVJ-load and DLVJ-land), loading phase of the SLS, and landing phase of the SLDL. The loading phases for DLVJ and SLS were defined as the period between initiation of knee flexion and the cessation of knee flexion. The landing phases for DLVJ and SLDL were defined as the period from initial foot contact to the cessation of knee flexion occurring immediately after flight. Phases of interest were determined visually for the 2D assessment. For 3D data, events were placed using a custom MATLAB (MATLAB 2020b, Natick, Massachusetts, USA) code at the time points of interest, including the time of initiation of knee flexion and the point of maximum knee flexion.

### 2.4 Data analysis

One physical therapist with seven years of clinical experience was trained on the four scoring criterion and independently scored all videos. Training was conducted prior to each scoring session, in which a single scoring criteria was reviewed and competency was assessed. A two-week washout period was allotted between each scoring method to reduce recall bias. For the 3D kinematic data, a custom MATLAB six degrees-of-freedom model was used to compute lower extremity joint angles. 3D data was extracted at the time point in which the knee joint center (midpoint between the medial and lateral femoral epicondyle) was most medial relative to the ankle joint center (midpoint between medial and lateral malleolus) during the descent phase (initiation of knee flexion to maximum knee flexion). This point of interest for lower extremity joint position was chosen in order to align with the time point in which the 2D data was scored. Coronal plane (frontal view) joint angles described in prior work by Hewett et al. as components of DKV were calculated, including pelvic obliquity, hip adduction, knee abduction, ankle eversion, and foot progression angle (10). Positive joint angles indicate hip adduction, knee valgus, and ankle eversion, while pelvic obliquity and foot progression angles are global measures relative to the lab coordinate system, with ipsilateral pelvic rise and internal foot progression values indicated as positive values.

### 2.5 Statistical analysis

The percentage of participants that exhibited a present injury risk factor according to each 2D scoring criterion was determined and the mean and standard deviation values for all 3D kinematic variables were calculated. Logistic regression analysis was used to determine whether 3D biomechanical variables were associated with the corresponding 2D scoring criterion. Specifically, a regression was performed for each biomechanical variable of interest from each of the three tasks (including two phases of the DLVJ) against all four 2D scoring criterion. If a significant association was observed, receiver operating characteristic (ROC) curves were created to determine 3D cut points to allow for better evaluation of each 2D scoring criterion in identifying participants who exhibited a movement fault. This cut point was identified such that the scoring criterion correctly identifies the greatest number of participants demonstrating a risk factor (true positives, measured via sensitivity) while minimizing the number of participants incorrectly identified for exhibiting an injury risk factor (false positives, measured via specificity) (51). Accuracy of each 2D criterion compared to 3D biomechanical measures was described by computing the area under the ROC curve (AUC), in addition to the sensitivity and specificity values, for each kinematic variable (i.e., pelvic obliquity, hip adduction, knee abduction, ankle eversion, and foot progression angle). Statistical significance was determined when *p *< 0.05 (R programming, version 4.3.0, R Development Core Team), and the AUC was used to classify the statistical model as outstanding (0.90–1.00), excellent (0.80–0.89), acceptable (0.70–0.79), poor (0.51–0.69), or no discrimination (0.50 or less) (52).

## 3 Results

A total of 41 adolescent female club volleyball participants (age: 14.7 ± 1.4 years; BMI: 21.5 ± 3.7) completed the three-task movement screen. Nine participants were removed from analysis due to errors made when completing one or more of the tasks. Frequency of present movement faults and mean (SD) biomechanical variables per task, extracted when the knee joint center was most medial, are presented in Tables 1, 2, respectively. Briefly, movement faults were identified as present most frequently with SHOE for DLVJ-Load (57.8%) and DLVJ-Land (68.8%; Table 1). The most movement faults were identified as present using LINE during the SLS (71.9%) and DIFF during the SLDL (59.4%). There were differences in 3D pelvic obliquity and hip adduction in the group identified to have a present 2D movement fault compared to those without a movement fault for the single-leg tasks. For DLVJ, ankle eversion was significantly different in the group with a present 2D movement fault, while differences in hip adduction was measured during DLVJ-Load only.

**Table 1:** Percent identified at risk (%) by 2D scoring criterion.

** **	MEDIAL	SHOE	LINE	DIFF
SLS	15.6	54.7	71.9	53.1
SLDL	9.4	48.4	50.0	59.4
DLVJ-Load	37.5	57.8	35.9	25.0
DLVJ-Land	54.7	68.8	45.3	50.0

**Table 2:** 3D biomechanical variables according to presence of 2D movement faults.

	MEDIAL		SHOE		LINE		DIFF	
SLS	Present	Not present	Present	Not present	Present	Not present	Present	Not present
Pelvic obliquity	**4.29** (**3.69)**	**−0.07** (**4.96)**	**1.94** (**4.69)**	**−0.98** (**5.01)**	**2.21** (**4.52)**	**−3.46** (**3.86)**	**2.41** (**4.39)**	**−1.42** (**4.98)**
Hip adduction	**19.10** (**6.73)**	**13.51** (**7.38)**	**16.73** (**6.86)**	**11.56** (**7.40)**	**16.62** (**6.99)**	**8.68** (**5.65)**	**17.63** (**7.09)**	**10.70** (**6.26)**
Knee valgus	2.61 (3.25)	2.33 (4.31)	2.38 (3.90)	2.36 (4.49)	2.19 (4.25)	2.84 (3.94)	2.70 (2.95)	2.00 (5.20)
Ankle eversion	**−4.15** (**2.05)**	**−0.08** (**3.12)**	−1.41 (3.11)	0.12 (3.43)	−1.22 (3.17)	0.59 (3.42)	−1.48 (3.08)	0.15 (3.41)
Foot progression	−5.61 (4.58)	−5.96 (5.24)	−6.95 (5.88)	−4.65 (3.70)	−5.14 (4.48)	−7.87 (6.16)	**−4.60** (**3.81)**	**−7.39** (**5.99)**
SLDL	Present	Not present	Present	Not present	Present	Not present	Present	Not present
Pelvic obliquity	**0.14** (**4.17)**	**−7.26** (**4.43)**	**−4.36** (**4.13)**	**−8.64** (**4.67)**	**−4.47** (**4.79)**	**−8.67** (**4.06)**	**−4.86** (**4.46)**	**−9.07** (**4.44)**
Hip adduction	**12.63** (**7.03)**	**−3.71** (**7.76)**	**1.66** (**9.14)**	**−5.78** (**7.37)**	**2.03** (**9.80)**	**−6.38** (**5.73)**	**0.62** (**9.83)**	**−6.26** (**5.73)**
Knee valgus	1.30 (7.42)	4.04 (3.25)	3.11 (4.59)	4.42 (2.84)	3.35 (4.49)	4.21 (3.01)	3.37 (4.10)	4.38 (3.35)
Ankle eversion	−2.11 (3.89)	−1.58 (3.96)	−2.14 (3.32)	−1.14 (4.41)	−2.28 (2.96)	−0.97 (4.65)	−2.38 (3.34)	−0.53 (4.49)
Foot progression	−3.38 (6.91)	−1.45 (5.78)	−1.53 (6.15)	−1.73 (5.66)	−0.67 (5.67)	−2.59 (5.97)	−1.37 (6.10)	−2.01 (5.57)
DLVJ-Load	Present	Not present	Present	Not present	Present	Not present	Present	Not present
Pelvic obliquity	0.50 (3.59)	−0.21 (1.51)	0.35 (3.05)	−0.33 (1.40)	0.61 (3.67)	−0.25 (1.47)	−0.11 (3.54)	0.12 (2.09)
Hip adduction	**−3.10** (**4.46)**	**−1.65** (**3.14)**	**−1.27** (**4.18)**	**−3.46** (**2.56)**	−1.29 (4.95)	−2.70 (2.77)	−0.31 (5.37)	−2.82 (2.79)
Knee valgus	4.19 (3.59)	3.74 (3.69)	4.20 (4.15)	3.51 (2.78)	3.84 (4.69)	3.95 (2.93)	4.62 (3.39)	3.68 (3.71)
Ankle eversion	**−15.5** (**3.34)**	**−10.1** (**2.67)**	**−13.11** (**3.82)**	**−10.78** (**3.73)**	**−14.66** (**3.85)**	**−10.70** (**3.22)**	**−14.04** (**4.51)**	**−11.49** (**3.54)**
Foot progression	−9.60 (6.18)	−9.48 (5.75)	−9.27 (6.07)	−9.87 (5.66)	−9.78 (5.99)	−9.38 (5.86)	−10.98 (8.19)	−9.04 (4.87)
DLVJ-Land	Present	Not present	Present	Not present	Present	Not present	Present	Not present
Pelvic obliquity	−0.25 (3.30)	−0.14 (2.29)	0.00 (3.10)	−0.64 (2.28)	0.16 (3.23)	−0.50 (2.53)	−0.27 (3.36)	−0.13 (2.33)
Hip adduction	−4.58 (5.04)	−3.90 (3.63)	−3.84 (4.95)	−5.23 (2.89)	−3.84 (5.06)	−4.63 (3.89)	−3.50 (5.27)	−5.05 (3.31)
Knee valgus	5.07 (3.36)	4.12 (3.20)	4.63 (3.34)	4.64 (3.30)	5.02 (3.44)	4.32 (3.19)	4.03 (2.94)	5.25 (3.56)
Ankle eversion	**−11.87** (**5.58)**	**−8.00** (**5.59)**	**−11.38** (**5.81)**	**−7.33** (**5.12)**	**−12.42** (**5.44)**	**−8.21** (**5.60)**	−11.37 (5.66)	−8.86 (5.90)
Foot progression	−9.60 (7.49)	−10.50 (7.48)	−9.91 (8.25)	−10.23 (5.45)	−8.60 (7.72)	−11.17 (7.10)	**−7.98** (**7.79)**	**−12.03** (**6.58)**

All variables are presented in degrees as Mean (SD). Positive values indicate ipsilateral pelvic rise, hip adduction, knee valgus, ankle eversion, and internal foot progression angle. Bold values indicate statistically significant differences (*p *< 0.05) between groups with a Present vs. Not Present 2D movement fault.

3D variables associated with DKV (i.e., components of DKV) resulted in AUCs between 0.62 and 0.93 across all scoring criterion and are presented in Table 3. The 2D scoring criterion with the greatest association to the components of DKV was MEDIAL with AUC values ranging from 0.67 to 0.93. Single-leg tasks resulted in the most statistically significant models relating 2D scoring criterion to 3D biomechanical measures of the pelvis and hip, while the double-leg task, across both the loading and landing phases, resulted in the most statistically significant models associated with the ankle. Notably, no 2D scoring criterion was able to identify 3D knee abduction.

**Table 3:** Receiver operating characteristic (ROC) curve analysis results by scoring criterion.

		MEDIAL					SHOE					LINE					DIFF				
		*p*-value	CP (°)	SP (%)	SE (%)	AUC	*p*-value	CP (°)	SP (%)	SE (%)	AUC	*p*-value	CP (°)	SP (%)	SE (%)	AUC	*p*-value	CP (°)	SP (%)	SE (%)	AUC
SLS	Pelvic obliquity	**0**.**018**	**0**.**2**	57.4	100.0	0.77	**0**.**024**	**−0**.**6**	58.6	77.1	0.67	**<0**.**001**	**1**.**3**	94.4	58.7	0.82	**0**.**004**	**2**.**3**	83.3	55.9	0.71
	Hip adduction	**0**.**041**	**18**.**1**	66.7	80.0	0.72	**0**.**008**	**16**.**9**	79.3	62.9	0.70	**0**.**001**	**9**.**4**	66.7	84.8	0.81	**0**.**001**	**18**.**7**	86.7	58.8	0.78
	Knee valgus	0.847					0.985					0.570					0.498				
	Ankle eversion	**0**.**002**	**1**.**8**	75.9	100.0	0.88	0.070					0.055					0.055				
	Foot progression angle	0.840					0.080					0.062					**0**.**037**	**−7**.**0**	53.3	79.4	0.67
SLDL	Pelvic obliquity	**0**.**013**	**−4**.**0**	81.0	100.0	0.93	**0**.**001**	**−5**.**8**	72.7	74.2	0.76	**0**.**002**	**−5**.**8**	75.0	75.0	0.78	**0**.**002**	**−4**.**4**	96.2	52.6	0.76
	Hip adduction	**0**.**003**	**1**.**2**	77.6	100.0	0.93	**0**.**002**	**−1**.**6**	87.9	61.3	0.75	**0**.**001**	**−3**.**5**	75.0	75.0	0.76	**0**.**005**	**−1**.**6**	88.5	52.6	0.71
	Knee valgus	0.116					0.178					0.370					0.301				
	Ankle eversion	0.750					0.309					0.188					0.072				
	Foot progression angle	0.443					0.896					0.192					0.666				
DLVJ-Load	Pelvic obliquity	0.276	* *	* *	* *	* *	0.288	* *	* *	* *	* *	0.194	* *	* *	* *	* *	0.750				
	Hip adduction	0.140					**0**.**027**	**−0**.**6**	92.6	37.8	0.66	0.157					**0**.**033**	**−0**.**6**	81.3	43.8	0.62
	Knee valgus	0.629					0.452					0.902					0.367				
	Ankle eversion	**<0**.**001**	**12**.**1**	77.5	87.5	0.91	**0**.**024**	**10**.**5**	59.3	78.4	0.68	**0**.**001**	**12**.**1**	73.2	82.6	0.80	**0**.**030**	**12**.**2**	64.6	75.0	0.68
	Foot progression angle	0.938					0.689					0.794					0.253				
DLVJ-Land	Pelvic obliquity	0.878	* *	* *	* *	* *	0.403	* *	* *	* *	* *	0.359	* *	* *	* *	* *	0.841	*** ***	* *	* *	* *
	Hip adduction	0.537					0.248					0.479					0.164				
	Knee valgus	0.254					0.995					0.396					0.143				
	Ankle eversion	**0**.**013**	**12**.**0**	82.8	60.0	0.71	**0**.**015**	**10**.**5**	80.0	59.1	0.70	**0**.**007**	**12**.**0**	80.0	65.5	0.73	0.094				
	Foot progression angle	0.629					0.871					0.174					**0**.**036**	**−8**.**3**	81.3	56.3	0.68

CP, cut point; SP, specificity; SE, sensitivity; AUC, area under the curve. AUC, sensitivity, and specificity are designated as outstanding (AUC ≥ 0.9, dark green), excellent (0.8 ≤ AUC < 0.9, light green), acceptable (0.7 ≤ AUC < 0.8; orange), and poor (0.5 ≤ AUC < 0.7; yellow). Positive CP values indicate ipsilateral pelvic rise, hip adduction, ankle eversion, and internal foot progression angle.

Cut points were identified for pelvic obliquity (SLS: −0.6° to 2.3°; SLDL: −4.0° to −5.8°) and hip adduction (SLS: 9.4° to 18.7°; SLDL: −3.5° to 1.2°) during the single-leg tasks (SLS and SLDL) for all scoring criterion, with sensitivity and specificity ranging from 55.9%–100.0% and 57.4%–96.2%, respectively (Table 3). During DLVJ-Load, a hip adduction cut point of −0.6° was identified for both SHOE (sensitivity 37.8%; specificity 92.6%) and DIFF (sensitivity 43.8%; specificity 81.3%). Ankle eversion cut points were identified for SLS using MEDIAL of 1.8° (sensitivity 100.0%; specificity 75.9%). For DLVJ-Load and DLVJ-Land, ankle eversion cut points were between 10.5° and 12.2°, with sensitivity and specificity ranging from 59.1%–87.5% and 59.3%–82.8%, respectively (Table 3). Lastly, DIFF was associated with foot progression angle cut points of −7.0° and −8.3° (external foot progression) during SLS and DLVJ-Land, respectively.

## 4 Discussion

The purpose of this study was to compare four different 2D scoring criterion for DKV based on analogous biomechanical variables captured through 3D motion capture. While no 2D scoring criterion successfully identified 3D knee abduction angle, other biomechanical components of DKV demonstrated significant AUC values compared to each 2D scoring criterion. Specifically, the MEDIAL 2D criterion provided the greatest agreement across tasks and the most consistent agreement across 3D biomechanical variables, with AUCs ranging from 0.67 to 0.93. Alternatively, LINE demonstrated the highest AUCs for pelvic obliquity and hip adduction (AUC 0.82 and 0.81, respectively). Using MEDIAL, cut points for were identified for pelvic obliquity and hip adduction during the single leg tasks, and for ankle eversion during the SLS. During the DLVJ, the best agreement was found for ankle eversion when utilizing MEDIAL, while the DIFF and SHOE identified cut points for hip adduction during the loading phase of the DLVJ.

The four movement fault criterion used in this study were derived based on findings of prior literature. Several studies have assessed 2D frontal plane knee motion by analyzing the angle formed by a line from the ASIS to the knee joint center and a line from the knee joint center to the ankle joint center (21, 40, 47, 48). This 2D criterion corresponds to the frontal plane progression angle (FPPA), which is analogous to LINE in the current study, and has been shown to demonstrate reliability and validity compared to 3D knee abduction during a SLS task (49). Discrepancy with the current study's findings is likely given that the LINE criterion adopted did not require identification of specific bony landmarks at the hip joint, such as the ASIS. With the goal of establishing a criterion that could easily be used on the field or in the clinic setting, a specific bony landmark was not chosen given that sufficient visualization of the pelvis and hip joints may not always be available, especially when instructed to place hands on hips.

In a study by Hewett et al., female athletes who subsequently experienced an ACL injury following biomechanical testing displayed 7.6° greater (3D) knee abduction range-of-motion compared to the remaining athletes who did not experience an ACL injury (39). Although Hewett and colleagues utilized a drop vertical jump task (dropping off from 30.5 cm high box), the concept of frontal plane knee displacement was employed as the basis for the DIFF scoring criterion, which evaluates whether the knee joint moves medially relative to the reference position of the lateral edge of the hip during single-leg tasks. Similarly, for the double leg vertical jump task, knee separation distance has been found to correlate to medial knee collapse and corresponds to DIFF in the current study (50). Lastly, medial knee displacement can also be examined by noting the knee position relative to the foot. Ageberg et al. found that participants who performed a single limb squat with a visually observed knee medial to foot position exhibited more medial thigh and tibia 2D angles compared to those who performed the squat with their knee over their foot in the frontal plane (47). Visual assessment of frontal plane knee motion using the knee relative to foot criterion was found to be valid compared to 2D knee “valgus” (2D angle between the thigh and shank); however, there was no relationship found with 3D knee abduction (47). MEDIAL and SHOE in the current study were derived similar to definitions using the position of the knee relative to the foot, and similar results were found.

In prior work, the author team also reported poor agreement for the knee position risk factor compared to 3D motion capture using the MEDIAL criterion (36), supporting the claim that 2D assessments of frontal plane measures alone do not reflect 3D knee biomechanics (19, 29, 48, 53). Previous studies have highlighted that cross-talk measured with 3D techniques result in inaccurate measurement of 3D knee abduction in tasks requiring greater amounts of knee flexion, resulting in the lack of agreement with 2D methods (33–35). Conflicting reports on the kinematic mechanisms of ACL injury exist; however, most agree that it is a result of a combination of movements and rotations of the hip, knee, and ankle, specifically hip adduction and internal rotation, knee abduction, tibial external rotation and anterior translation, and ankle eversion (37). Therefore, biomechanical contributors to dynamic knee valgus, such as hip adduction, pelvic drop, and foot position, might better align with 2D visual assessment of faulty movement patterns. For example, Padua et al. found that poor LESS scores were associated with increased 3D hip adduction during a drop vertical jump (23). Interestingly, differences between the “poor” and “excellent” groups were less than 1° of hip adduction, which may not be a clinically relevant difference. The group with “excellent” LESS scores had 0.7° of peak hip adduction compared to 1.6° in the “poor” group. While the DLVJ task in the current study is a double leg vertical jump, it is initiated from the floor as opposed to jumping off a platform as with the drop vertical jump, and is not directly comparable to the protocol employed in the Padua paper. In the current study, cut points for hip adduction were identified during the DLVJ-Load with SHOE and DIFF, indicating −0.6°, while higher cut points were established in the single leg tasks, ranging from 9.4°–18.7° and −3.5° to 1.2° during the SLS and SLDL, respectively. During DLVJ-Load and DLVJ-Land, our 3D data illustrated differences around 2 degrees in hip adduction between the group with a present 2D movement fault compared to those without a movement fault using SHOE and DIFF. While the SHOE criteria demonstrated statistically significant differences between groups during DLVJ-Load, 2 degrees is not typically considered to be a clinically relevant finding for 3D biomechanical data. Given greater hip adduction is likely in single-leg tasks as the center of mass shifts toward the stance limb, these cut points may prove clinically problematic for SHOE and DIFF as these criteria appear to be less sensitive in identifying this component of DKV.

Similar to the LESS tool, Herrington et al. investigated the validity of a qualitative screening for the single leg squat and single leg landing comprised of dichotomous scoring of the arms, trunk, pelvis, thigh, knee, and foot (28). 3D qualitative data were reduced into dichotomous scores based on previously published ranges for normative data and strong agreement was found between 3D and 2D scores, specifically 100% agreement for pelvic obliquity and hip adduction (28). While the methodology differs in this study, the ability for 3D measures to show excellent association with 2D assessment of trunk and lower limb alignment (specifically pelvis and hip position) during two single leg loading tasks supports the utility of 2D movement assessments for screening purposes. Although 3D knee abduction may not be evaluated via 2D methods, other components of DKV, and thus ACL injury risk, may be sufficient for identifying adolescent athletes requiring intervention.

Ankle motion has also been investigated as a component of DKV. More specifically, ankle eversion, in combination with external tibial rotation, increases torque at the knee which can contribute to injury (37). Several authors have shown that excessive ankle eversion results in increased valgus knee stress, anterior tibial translation, and increased loading on the ACL (54, 55). In a study by Kagaya et al. that tested 130 adolescent female basketball athletes, greater “knee-in distance” (distance between hallux and intersection of a line from the patella and ASIS to floor) during both a single leg squat and single leg landing task was associated with the presence of rear-foot eversion greater than 5° (56). While Kagaya and colleagues utilized a similar 2D analysis technique and evaluated lower extremity alignment at maximum medial knee position, the criterion employed to assess knee position (i.e., knee-in distance) was unlike the criterion used in this study.

When considering a double leg task, Chun et al. identified that a population of non-athletic collegiate females performed a drop vertical jump with reduced ankle eversion compared to male participants, with peak eversion of 2° over the landing cycle (57). Chun et al. evaluated a college-aged non-athletic population and though the difference observed was similar to the cut point indicated in the current study for ankle eversion by the MEDIAL criterion in this study, their findings may not be generalizable to an adolescent athlete population. Lastly, in a study by Ford et al. (16), kinematics during an unanticipated cutting maneuver were evaluated and adolescent female athletes demonstrated increased maximum eversion of 19.7° compared to males, which was interpreted by the authors as increased ACL injury risk in the female cohort. In this study, we were able to identify a cut point of 12.1° of eversion during DLVJ-Load and 12.0° during DLVJ-Land with MEDIAL and LINE with excellent to outstanding AUCs during loading and acceptable AUCs during landing. Thus, given similar thresholds of risk reported in prior literature, the cut points identified within our dataset of 12° of ankle eversion for the double leg task and 2° during the SLS with MEDIAL are appropriate.

### 4.1 Limitations

This study has several limitations. First, the results may not be generalizable to sports other than volleyball or to male athletes. Future research should be conducted to further validate the described movement screen in male athletes and across other sports. Second, rotational components of dynamic knee valgus were not considered due to the inherent limitations of 2D movement assessments. Internal rotation of the hip, combined with external tibial rotation, have been identified as contributing factors to the ACL injury mechanism (37). Although 2D movement screens have limitations in assessing rotation, the ability to employ them on a broad and cost-effective scale should be considered against availability of certain data points (e.g., rotational movement faults). Additionally, while 2D movement screens are more accessible, the assessment might be too time consuming in certain clinical settings. Third, the presence of retroreflective markers during 2D video analysis may have influenced the rater in identification of bony landmarks and subsequent identification of movement faults for one or more scoring criterion. Finally, 2D scoring was completed by a single rater, therefore the data relied exclusively on one scorer's evaluation. Future work will investigate inter- and intra-rater reliability utilizing the preferred scoring criteria.

## 5 Conclusions

Overall, the findings presented in this study demonstrate the utility of a 2D assessment of coronal plane movement faults compared to 3D biomechanics in adolescent female volleyball players. Among four unique criteria, the MEDIAL criterion demonstrated the best performance in distinguishing components of DKV, specifically pelvic obliquity, hip adduction, and ankle eversion, for a three-task movement screen involving both single- and double-leg tasks. Along with the previously identified scoring criterion for trunk-specific movement faults, the authors suggest that the MEDIAL criterion may be the most indicative of DKV—an ACL injury risk factor—given an association with 3D biomechanical risk factors. This work provides an accessible tool to identify faulty movement patterns among adolescent athletes.

## Data Availability

The raw data supporting the conclusions of this article will be made available by the authors, without undue reservation.
